# Immune landscape of breast tumors with low and intermediate estrogen receptor expression

**DOI:** 10.1038/s41523-023-00543-0

**Published:** 2023-05-13

**Authors:** Leonie Voorwerk, Joyce Sanders, Milou S. Keusters, Sara Balduzzi, Sten Cornelissen, Maxime Duijst, Esther H. Lips, Gabe S. Sonke, Sabine C. Linn, Hugo M. Horlings, Marleen Kok

**Affiliations:** 1 Division of Tumor Biology & Immunology, Netherlands Cancer Institute, Amsterdam, the Netherlands; 2 Department of Pathology, Netherlands Cancer Institute, Amsterdam, the Netherlands; 3 Department of Biometrics, Netherlands Cancer Institute, Amsterdam, the Netherlands; 4 Core Facility Molecular Pathology & Biobanking, Netherlands Cancer Institute, Amsterdam, the Netherlands; 5 Division of Molecular Pathology, Netherlands Cancer Institute, Amsterdam, the Netherlands; 6 Department of Medical Oncology, Netherlands Cancer Institute, Amsterdam, the Netherlands; 7 Department of Pathology, University Medical Center Utrecht, Utrecht, The Netherlands

**Keywords:** Breast cancer, Tumour immunology, Cancer

## Abstract

Immune checkpoint blockade (ICB) is currently approved for patients with triple-negative breast cancer (TNBC), whereas responses to ICB are also observed in a small subgroup of Estrogen Receptor (ER)-positive breast cancer. The cut-off for ER-positivity (≥1%) is based on likelihood of endocrine treatment response, but ER-positive breast cancer represents a very heterogeneous group. This raises the question whether selection based on ER-negativity should be revisited to select patients for ICB treatment in the context of clinical trials. Stromal tumor-infiltrating lymphocytes (sTILs) and other immune parameters are higher in TNBC compared to ER-positive breast cancer, but it is unknown whether lower ER levels are associated with more inflamed tumor microenvironments (TME). We collected a consecutive series of primary tumors from 173 HER2-negative breast cancer patients, enriched for tumors with ER expression between 1 and 99% and found levels of stromal TILs, CD8 + T cells, and PD-L1 positivity in breast tumors with ER 1–9% and ER 10–50% to be comparable to tumors with ER 0%. Expression of immune-related gene signatures in tumors with ER 1–9% and ER 10–50% was comparable to ER 0%, and higher than in tumors with ER 51–99% and ER 100%. Our results suggest that the immune landscape of ER low tumors (1–9%) and ER intermediate tumors (10–50%) mimic that of primary TNBC.

## Introduction

Estrogen receptor (ER) expression assessment is one of the cornerstones in the diagnostic work-up for breast cancer and is an essential biomarker for prediction of endocrine treatment efficacy^1,2^. Current ASCO/CAP recommendations define ER-positive tumors as having ≥1% ER expression and tumors with ER expression between 1 and 10% as ER low-positive tumors^2^. This cut-off is based on studies that reported lack of responses to endocrine treatment in tumors with no ER expression^3,4^, but a pragmatic cut-off of 10% is sometimes used in clinical trials^5–8^ and in daily practice^9^. Although the cut-off was originally meant for endocrine treatment response, it is also being used for selecting breast cancer for novel treatments such as immune checkpoint blockade (ICB). Approximately 2–5% of HER2-negative patients have breast cancer with low-positive ER (1–9%) expression^10–12^. Recently, several studies demonstrated that patients with ER low-positive HER2-negative breast cancer have similar outcomes as compared to triple-negative breast cancer (TNBC) patients^10–14^. ER low-positive breast tumors have comparable progesterone receptor (PR) levels^12,15^, tumor grade^10–13,16^ and Ki-67 expression^11,13,16^ to TNBC and are usually classified as basal-like or HER2-enriched^11,15,17^. Intermediate ER expression of 10–50% is common in ~5–10% of breast tumors^18,19^ and we hypothesize that this group might share basal-like features with TNBC similar to the ER low-positive group.

In general, as compared to ER-positive tumors, ER-negative tumors have a more inflamed tumor microenvironment (TME), characterized by prominent stromal tumor-infiltrating lymphocyte (sTIL) infiltration^20^, CD8 + T-cells^21^ and higher expression of immune-related gene sets^22^. sTILs and CD8 + T cells are positively associated with prognosis and chemotherapy efficacy in early-stage TNBC^20,21,23–26^, while in ER-positive breast cancer the role of immune cell infiltration is less clear^20,27,28^. Responses to ICB are also more prominent in patients with TNBC^29^. Neo-adjuvant ICB plus chemotherapy is currently approved for early-stage TNBC^30^ and promising results have been observed in high-risk ER-positive breast cancer^8,31,32^. Exploratory biomarker studies from these and other trials demonstrated that expression of immune-related genes are associated with response^33^ or survival^34^ to neo-adjuvant ICB in early-stage TNBC, while sTILs and PD-L1 expression mainly have predictive value in the metastatic setting^35,36^. It is currently not known whether breast tumors with low-positive (1–9%) or intermediate-positive (10–50%) ER expression are comparable to TNBC in terms of immune characteristics that are relevant for ICB response and whether these patients are therefore more likely to respond to ICB.

In this study, we aim to explore immunological characteristics of HER2-negative breast tumors with low-positive (1–9%) or intermediate-positive (10–50%) ER expression, as compared to TNBC and tumors with high ER expression (>50%). Using a consecutive series of tumor blocks, enriched for tumors with ER expression between 1 and 99%, we investigated clinicopathological characteristics and features of the TME that have previously been associated with response to ICB in breast cancer.

## Results

### Clinicopathological characteristics in relation to ER expression levels

A series of tumor blocks from 173 HER2-negative patients was collected, enriched for ER expression between 1–9%, 10–50% and 51–99%. All patients diagnosed in the Netherlands Cancer Institute between 2011 and 2019 with HER2-negative primary breast cancer with ER expression between 1 and 50% and for whom tumor material was available were identified, of which 17 patients had tumors with ER1-9% (low-positive) and 22 patients had tumors with ER10-50% (intermediate-positive; Supplementary Fig. 1). Subsequently, a consecutive series of tumors with ER0% (negative; *n* = 46), ER51-99% (high; *n* = 37) and ER100% (ultrahigh; *n* = 51) within these diagnosis years were collected, aiming for balanced group sizes. For each patient, an in-house tumor block of a pre-treatment biopsy (in case of neo-adjuvant treatment) or resection was collected. We observed slight differences in tumor size and nodal stage between the groups, with the highest proportion of small tumors within the ER100% group and the highest proportion of lymph node-negative patients in the group with ER1-9% (Table 1). Four patients had a germline *BRCA1* mutation within the ER0% group and three patients had a germline *BRCA2* mutation within the ER100% group. As expected, ER expression highly correlated with PR expression and negatively correlated with tumor grade and Ki67 levels (Table 1, Supplementary Fig. 2A-C). In the groups with low-positive and intermediate-positive ER expression we observed a lower proportion of grade 3 tumors and lower Ki-67 expression levels as compared to ER-negative tumors, but a higher proportion of grade 3 tumors as compared to the groups with high ER expression (>50%; Supplementary Fig. 2B, C).

**Table 1:** Patient characteristics.

*N* = 173 No. of patients (%)		ER 0% (*n* = 46)	ER 1–9% (*n* = 17)	ER 10–50% (*n* = 22)	ER 51–99% (*n* = 37)	ER 100% (*n* = 51)	*P* value
Age	Median (range)	55 (26–79)	64 (35–89)	56 (38–84)	54 (28–82)	59 (31–80)	0.26
	≤50	21 (46)	7 (41)	8 (36)	18 (49)	19 (37)	0.80
	>50	25 (54)	10 (59)	14 (64)	19 (51)	32 (63)	
	≤60	29 (63)	7 (41)	12 (55)	27 (73)	29 (57)	0.22
	>60	17 (37)	10 (59)	10 (45)	10 (27)	22 (43)	
Menopausal status	Pre/peri	18 (39)	5 (29)	6 (27)	17 (46)	17 (33)	0.60
	Post	27 (59)	10 (59)	11 (50)	15 (41)	29 (57)	
	Unknown/NA	1 (2)	2 (12)	5 (23)	5 (14)	5 (10)	
Tumor stage	T1	17 (37)	11 (65)	15 (68)	20 (54)	38 (75)	0.01
	T2	24 (52)	6 (35)	5 (23)	15 (41)	12 (24)	
	T3	5 (11)	0 (0)	2 (9)	2 (5)	1 (2)	
Nodal stage	N0	36 (78)	15 (88)	15 (68)	22 (59)	39 (76)	0.09
	N1	7 (15)	2 (12)	3 (14)	13 (35)	11 (22)	
	N2-N3	3 (6)	0 (0)	4 (18)	2 (5)	1 (2)	
g*BRCA* mutation	*BRCA1*	4 (9)	0 (0)	1 (5)	0 (0)	0 (0)	0.07
	*BRCA2*	0 (0)	0 (0)	0 (0)	0 (0)	3 (6)	
	No mutation	29 (63)	10 (59)	10 (45)	14 (38)	13 (25)	
	Unknown	13 (28)	7 (41)	11 (50)	23 (62)	35 (69)	
PR expression	PR 0%	39 (85)	10 (59)	7 (32)	6 (16)	9 (18)	<0.0001
	PR1-9%	7 (15)	7 (41)	4 (18)	3 (8)	5 (10)	
	PR ≥ 10%	0 (0)	0 (0)	11 (50)	28 (76)	37 (73)	
Tumor grade	Grade 1	0 (0)	2 (12)	2 (9)	9 (24)	13 (25)	<0.0001
	Grade 2	2 (4)	4 (24)	11 (50)	22 (59)	31 (61)	
	Grade 3	44 (96)	9 (53)	7 (32)	6 (16)	7 (14)	
	Unknown	0 (0)	2 (9)	2 (9)	0 (0)	0 (0)	
Ki-67 expression	Ki-67 < 20%	4 (9)	7 (41)	11 (50)	27 (73)	37 (73)	<0.0001
	Ki-67 ≥ 20%	42 (91)	10 (59)	11 (50)	10 (27)	14 (27)	

Difference between groups was tested by Fisher’s exact test with excluded missing values. The median difference between age was tested by Kruskal–Wallis.

*NA* Not applicable, *gBRCA* germline BRCA.

### sTILs, CD8 + T cells and PD-L1 expression of tumors with low-positive and intermediate-positive ER expression

**Fig. 1:**
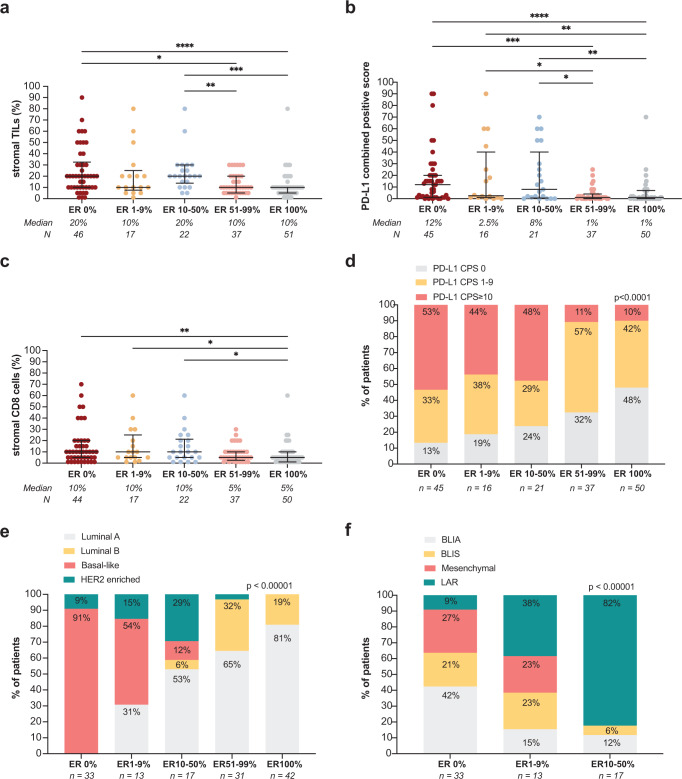
Immune cell composition in relation to estrogen receptor (ER) expression levels. **a** Levels of stromal tumor-infiltrating lymphocytes (sTILs) in relation to ER expression. **b** PD-L1 expression (clone 22C3), assessed as combined positive score (CPS) in relation to ER expression. PD-L1 staining was unavailable for 5 patients. **c** Levels of stromal CD8 + T cells (percentage of CD8 + T cells of the stromal area) in relation to ER expression. CD8 staining was unavailable for 3 patients. **d** Proportion of patients with PD-L1 positive tumors (CPS) using different cut-offs (0%, 1–9%, ≥ 10%) in relation to ER expression. **e** Proportion of patients with luminal A, luminal B, basal-like and HER2 enriched tumors in relation to ER expression. Molecular subtypes were assessed according to PAM50 with the NanoString nCounter® Breast Cancer 360™ panel. **f** Proportion of patients with basal-like immune activated (BLIA), basal-like immune-suppressed (BLIS), mesenchymal or luminal androgen receptor (LAR) tumors in relation to ER expression. TNBC subtypes were assessed with the NanoString nCounter® Breast Cancer 360™ panel. **a**–**c** Error bars display the median with interquartile range, statistics by Kruskal–Wallis with post-hoc Dunn’s test. Only statistically significant comparisons are shown. *p < 0.05, **p < 0.01, ***p < 0.001, ****p < 0.0001. **d**–**f** Numbers display percentage per group, statistics by Fisher’s exact test.

First, we assessed immune cell composition by investigating sTILs, stromal CD8 + T-cells and PD-L1 expression (assay 22C3, combined positive score). We observed highest levels of sTILs and continuous PD-L1 expression in the ER0% and ER10-50% groups, followed by the ER1-9% group (Fig. 1a, b). Median CD8 + T-cell levels were equal in the groups with ER0%, ER1-9% and ER10-50%, and higher as compared to the groups with ER51-99% and ER100% (Fig. 1c). Next, we assessed the proportion of PD-L1 positive tumors in the different groups using a cut-off of ≥ 1% and ≥ 10%. We observed that 86%, 82% and 77% of patients with ER0%, ER1-9% and ER10-50%, respectively, had PD-L1 positive tumors using a 1% cut-off, whereas this was only 68% and 52% for the groups with ER51-99% and ER100% (Fig. 1d). The same patterns were observed using the higher PD-L1 cut-off of 10%, with ~40–50% of tumors within the ER0%, ER1-9% and ER10-50% groups and only 11% and 10% with ER51-99% and ER100%, respectively, being PD-L1 positive (Fig. 1d). Investigating sTILs, CD8 + T-cell levels and PD-L1 expression in relation to age and menopausal status, we observed slightly higher sTIL levels in younger patients (Supplementary Fig. 3A–F). Higher PD-L1 expression and only slightly higher sTILs and CD8 + T-cell levels were seen in grade 3 tumors or tumors with high Ki-67 expression (Supplementary Fig. 3G–L), suggesting that these features are mainly associated with ER expression and may play a less dominant role in immune cell composition. Altogether, these data demonstrate that breast tumors with low-positive (1–9%) and intermediate-positive (10–50%) ER expression are comparable to ER-negative tumors in terms of sTILs, CD8 + T cells and PD-L1 expression.

### Intrinsic molecular subtypes of tumors with low-positive or intermediate-positive ER expression

As a basal-like molecular subtype has been described as possibly predictive of ICB response^8,37^, we assessed PAM50 subtypes (NanoString)^38,39^. We observed basal-like tumors in the groups with ER expression of 0% (91% of total), 1–9% (54% of total) and 10–50% (12% of total), but not in the groups with higher ER expression (Fig. 1e). In the low-positive and intermediate-positive ER groups, 31% and 59% of tumors, respectively, were classified as luminal A or B, as compared to 97% in the ER-high and 100% in the ER-ultrahigh groups, underlining the heterogeneous nature of the groups with ER expression between 1 and 50% (Fig. 1e). Next, we assessed the TNBC subtypes by Burstein et al.^40^ in the ER0%, ER1-9% and ER10-50% groups, as a basal-like immune-activated (BLIA) phenotype has been associated with response to ICB as well^37^. In our dataset, tumors with a BLIA phenotype were restricted to ER expression of 50% or lower. Interestingly, 15% and 12% of tumors with ER1-9% and ER10-50% expression, respectively, were classified as BLIA, as compared to 42% of tumors with ER0% (Fig. 1f). PD-L1 expression was highest in the BLIA tumors (Supplementary Fig. 3M). These findings demonstrate that within the breast cancer groups with low-positive or intermediate-positive ER expression, a subset of tumors is inflamed and exhibits molecular features of TNBC.

### Higher expression of immune-related genes in ER-negative, low-positive, and intermediate-positive tumors, as compared to ER high-positive tumors

**Fig. 2:**
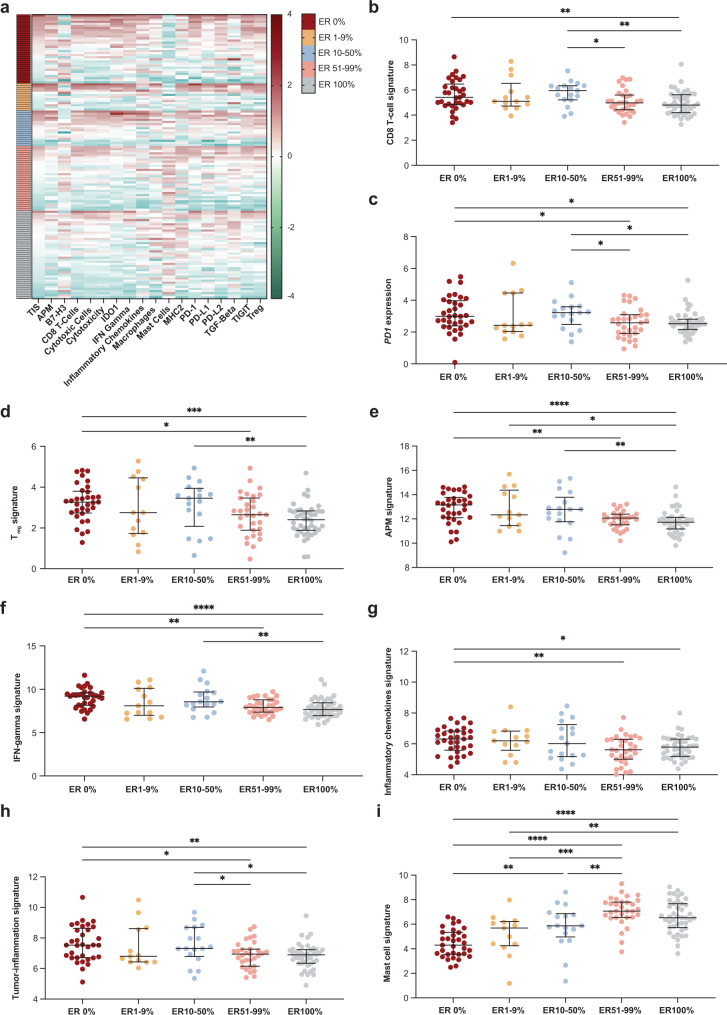
Expression of immune signatures in relation to estrogen receptor (ER) expression levels. **a** Heatmap of expression of immune signatures (*z*-scores), grouped by ER expression and sorted by tumor-inflammation signature (TIS)^64^ per group. **b** CD8 + T-cell signature expression in relation to ER expression. Genes included: *CD8A, CD8B*. **c** *PD1* expression in relation to ER. **d** Regulatory T-cell (T_reg_) signature in relation to ER expression. Genes included: *FOXP3*. **e** Antigen presenting machinery (APM) signature expression in relation to ER. Genes included: *TAP1, TAP2, TAPBP, HLA-A, HLA-B, HLA-C*. **f** Interferon (IFN)-γ signature expression in relation to ER expression. Genes included: *CXCL9, CXCL10, STAT1*. **g** Inflammatory chemokine signature expression in relation to ER expression. Genes included: *CCL2, CCL3L1, CCL4, CCL7, CCL8*. **h** TIS in relation to ER expression. Genes included: *CCL5, CD27, CD274, CD276, CD8A, CMKRL1, CXCL9, CXCR6, HLA-DQA1, HLA-DRB1, HLA-E, IDO1, LAG3, NKG7, PDCD1LG2, PSMB10, STAT1, TIGIT*. **i** Mast cell signature expression in relation to ER expression. Genes included: *MS4A2, CPA3, HDC, TPSAB1*. **b**–**i** Median with interquartile range, statistics by Kruskal–Wallis with post-hoc Dunn’s test. Only statistically significant comparisons are shown. *p < 0.05, **p < 0.01, ***p < 0.001, ****p < 0.0001.

To gain more insight in the immune biology of tumors with low-positive or intermediate-positive ER expression, we next analyzed expression of immune signatures using the NanoString nCounter® Breast Cancer 360™ panel. Within each ER subgroup there was a wide range of expression of all immune signatures, but in general immune signatures were most highly expressed in ER0%, ER1-9% and ER10-50% (Fig. 2a). Zooming in on the signatures that were significantly different between groups, we observed highest median levels of the CD8 + T cell signature, *PD1* mRNA expression and the regulatory T-cell (T_reg_) signature in the group with ER10–50%. These levels were not significantly different from the groups with ER1-9% and ER0%, but significantly higher as compared to the ER51-99% and/or ER100% groups (Fig. 2b–d). Median expression levels of signatures reflecting antigen presenting machinery (APM), IFN-γ signature, inflammatory chemokines and tumor-inflammation score (TIS) were all highest in breast tumors with no ER expression, not significantly different from the groups with low- or intermediate-positive ER expression and statistically significantly higher when comparing to the ER51-99% or ER100% groups (Fig. 2e–h). Mast cells were the only immune cells that were more abundantly present in the TME of ER-positive tumors, increasing with ER expression (Fig. 2a, i). Using pre-treatment gene expression data from an independent validation cohort of stage I-III breast cancer patients treated with neo-adjuvant chemotherapy^41^, we also observed higher levels of the CD8 + T-cell signature and IFN-γ signature in breast tumors with 0%, 1–9% and 10–50% ER expression, as compared to tumors with ER expression >50% (Supplementary Fig. 4A–D). In addition, also in this cohort we observed higher expression of mast-cell related genes in ER high-positive tumors, as compared to ER-negative or ER low-positive tumors (Supplementary Fig. 4E, F).

**Fig. 3:**
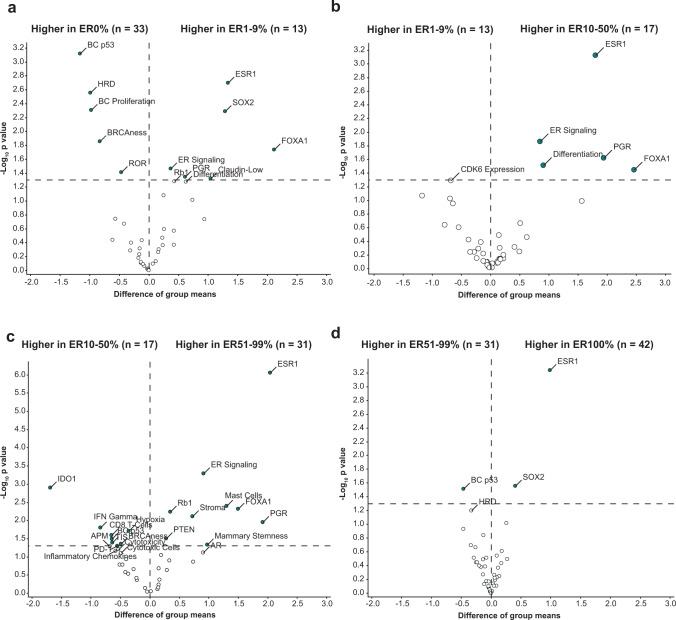
Differential gene expression of NanoString Breast Cancer 360™ signatures. **a** Difference in gene expression of signatures between the group with 0% ER expression and 1–9% ER expression. **b** Difference in gene expression of signatures between the group with 1–9% ER expression and 10–50% ER expression. **c** Difference in gene expression of signatures between the group with 10–50% ER expression and 51–99% ER expression. **d** Difference in gene expression of signatures between the group with 51–99% ER expression and 100% ER expression. **a**–**d** On the *x*-axis the difference in group means is displayed, on the *y*-axis the unadjusted *p* value per variable by student *t* tests. The vertical line indicates no change, the horizontal line indicates a *p* value of 0.05.

To assess transcriptomic differences including non-immune-related processes in relation to ER expression, we analyzed all 42 signatures of the NanoString panel and started with unbiased clustering of the tumors. Using a principal component analysis, we observed that tumors with 0% ER expression tended to cluster away from breast tumors with >50% ER expression, but tumors with ER1-9% and ER10-50% seemed to mix between the tumors with 0% and >50% ER expression (Supplementary Fig. 5A). With unsupervised clustering, two clusters were dominated by ER0% tumors and also included ER1-9% and ER10-50% tumors, which were characterized by either high expression of immune signatures or by genomic instability (Supplementary Fig. 5B). Comparing expression of each signature between the groups with ER0% and ER1-9%, we saw significantly lower expression of signatures characterizing genomic instability and p53 biology and, as expected, higher expression of ER-related signaling in the group with ER1-9% expression, but no significant differences in immune signatures (Fig. 3a). Investigating differential expression between tumors with ER10-50% and ER1-9%, we observed higher expression of ER signaling in the ER10-50% group, but again no significant differences in immune signatures (Fig. 3b). Comparing ER51-99% tumors with the group of ER10-50%, we observed higher expression of immune pathways in the tumors with ER expression between 10 and 50% and lower expression of ER signaling and mast cells (Fig. 3c). Between the groups with ER51-99% and ER100%, mainly a difference in *ESR1* expression was seen (Fig. 3d). To increase statistical power, we pooled the ER low-positive and intermediate-positive groups (ER1-50%) and confirmed our findings (Supplementary Fig. 6A, B). In summary, we observed that the expression of immune signatures was significantly higher in tumors with negative and low-positive or intermediate-positive ER expression as compared to tumors with high-positive ER expression, in line with our data on sTILs, CD8 + T cells and PD-L1 expression. Comparing to ER-negative tumors, we observed that ER low-positive and intermediate-positive breast tumors differ in ER signaling and genomic instability, but not in expression in immune pathways.

## Discussion

Breast cancer is a heterogeneous disease with ER expression being one of the most widely used biomarkers. The current cut-off of ≥ 1% for positive ER expression is based on early studies in which no benefit was seen with endocrine treatment in patients with no ER expression. Consequently, most translational studies and clinical trials focus on differences between TNBC and ER-positive breast cancer, with no further distinction in ER-positive breast cancer. In this study, we explore the immune landscape of early-stage breast tumors with different levels of ER expression. We are, to our knowledge, the first to demonstrate that early-stage breast tumors with low-positive (1–9%) and intermediate-positive (10–50%) ER expression have more similarities in immune biology to TNBC than to their highly ER-positive counterparts, based on sTILs, CD8 + T-cell presence, PD-L1 expression and expression of immune pathways. Our data highlight that clinical trials investigating ICB in ER-positive breast cancer should consider efficacy analysis in subgroups of patients with low-positive or intermediate-positive breast cancer.

Two phase II trials have reported results of neo-adjuvant ICB plus chemotherapy in high-risk ER-positive breast cancer. In the I-SPY2 trial, two arms with ICB-combinations graduated: pembrolizumab plus chemotherapy (taxane, followed by AC)^31^ and durvalumab/olaparib plus chemotherapy^32^. In both arms, it was shown that pCR rates were higher in the experimental arms as compared to the control arms in the high-risk (based on MammaPrint) ER-positive subgroup. Importantly, in an exploratory analysis, patients with an ultrahigh MammaPrint signature derived most benefit from the addition of durvalumab/olaparib to chemotherapy, which correlated with low *ESR1* and *PGR* expression and high proliferation^32^. In addition, low *ESR1* and *PGR* expression was associated with higher pCR rates in the pembrolizumab arm^42^. In the single-arm GIADA-trial, a pCR rate of 15% was observed after neo-adjuvant chemotherapy (AC) plus nivolumab and exemestane in high-risk ER+ ( ≥ 10%) breast cancer, defined by high Ki-67 expression and/or tumor grade 3^8^. In this trial, a basal-like subtype highly correlated with response^8^. In addition, in metastatic ER-positive breast cancer, estrogen signaling was negatively associated with response to pembrolizumab and eribulin^43^. Results of these trials indicate that ICB responses are not limited to TNBC and that exploratory subgroup analysis of response in clinical trials investigating ICB in ER-positive breast cancer are of great importance to confirm the association between low and intermediate ER expression levels and ICB response.

While sTILs and CD8 + T cells are positively associated with outcome in early-stage TNBC^20,21,23–26^, this association is not clear in ER-positive breast cancer^20,27,28^, suggesting that T-cell functioning is hampered in ER-positive tumors. Several immune cells, particularly myeloid cells, express ER^44^. Estrogen signaling has been shown to increase mobilization and immune-suppressive functions of myeloid-derived suppressor cells (MDSCs) in vivo which drives disease progression^45^. Moreover, a recent study demonstrated that estrogen signaling in a murine melanoma model promoted the accumulation of immune-suppressive macrophages in the TME, reduced cytotoxicity of CD8 + T cells and promoted tumor growth^46^. Interestingly in this model, this detrimental effect could be reverted by treatment with fulvestrant and ICB^46^. In turn, immune signaling, via interferons and STAT1, has been implicated in increased transcription of ER in tumor cells^47^, indicating a positive feedback loop between estrogen and interferon signaling and highlighting the complex crosstalk between immune cells in the TME and ER signaling. In breast cancer, high levels of tumor-associated macrophages (TAMs) and mast cells with pleiotropic functions have been described^48,49^. Mast cells are more abundant in the TME of luminal breast cancers, as compared to basal-like tumors^50^ and have been correlated to residual disease after neo-adjuvant chemotherapy^51^ and a non-pCR in the pembrolizumab and durvalumab/olaparib arm in the ER-positive subgroup of the I-SPY2 trial^32,42^. In our study we observed high levels of mast cell-related gene expression in ER-high tumors as compared to TNBC or ER low/intermediate tumors. Interestingly, we found non-significant lower levels of sTILs, PD-L1 expression and some immune-related gene sets in the group with ER levels between 1-9% as compared to the group with ER levels 10–50% (Figs. 1a, b, 2a–f), suggesting that there might not be a linear correlation between ER and immune infiltrate. As also highlighted by our analysis on breast cancer signatures in these groups, tumors with ER expression between 1 and 50% comprise a heterogenous group in terms of underlying biology. Altogether, we hypothesize that higher levels of ER signaling might shape the TME of ER-positive breast cancer potentially promoting an immune-suppressive state, but it remains to be determined whether this only holds true for a certain level of ER expression (e.g., >50%).

Subgroup analysis in ongoing phase III trials testing neo-adjuvant ICB-chemotherapy in high-risk ER-positive breast cancer (NCT03725059, NCT04109066) and future trials are needed to validate whether patients with low-positive or intermediate-positive ER expression derive more benefit from ICB than patients with high expression of ER. Since responses to endocrine treatment have been observed in some patients with low-positive ER tumors^52^ and CDK4/6 inhibitors have clinical activity in patients with intermediate-positive ER tumors (<50%) albeit to a lesser extent than patients with ER-high tumors^53^, it remains to be determined what the optimal (combination) treatment regimen is. Ideally, a basket trial specifically for patients with low- and/or intermediate-positive breast cancer could provide answers on this question, testing neo-adjuvant ICB-combinations such as anti-PD1 plus endocrine treatment or ICB with other immuno-oncology agents such as anti-CTLA4 plus anti-PD1^54^. In our cohort, we did not observe higher levels of sTILs, CD8 + T cells or PD-L1 expression in grade 3 or highly proliferative (Ki-67 expression ≥ 20%) tumors with ER expression >50% (data not shown), suggesting that patients most likely to respond to CDK4/6 inibitors^53,55^ don’t have particularly immunogenic tumors. This is important in light of the substantial toxicity that has been observed with anti-PD1 plus CDK4/6 inhibitors^56^. Recently, Wolf et al. proposed a novel model on the redefinition of early-stage breast cancer subtypes based on the pathological response to targeted agents or ICB. Within the ER-positive HER2-negative immune-enriched subtype, an estimated pCR rate to neo-adjuvant pembrolizumab plus chemotherapy of 69% was seen^57^. Based on our data, we hypothesize that this immune-enriched subtype might mostly be comprised of breast tumors with low-positive or intermediate-positive ER expression.

Our study is limited by its small sample size of the ER low-positive and intermediate-positive groups, although this is inherent to the relatively low incidence of these breast cancers in a single centre and the lack of reporting of continuous ER expression in most cancer registries and pathology laboratories. Furthermore, it has been proposed that low-positive ER tumors are an artefact of a low intensity staining^58^. However, in our study all ER stainings were done in concordance with Dutch guidelines for breast cancer diagnostics^9^ in one expert centre laboratory, including both internal controls and control tissues to ensure accurate receptor staining and were scored by dedicated breast pathologists. Third, we collected tumor blocks of the ER-negative and the two high-positive groups in a short consecutive series to roughly match the group size of the pooled ER low-positive and intermediate-positive group. This series was not matched in terms of TNM stage, resulting in slightly unbalanced T-stage and N-stage between groups. Since sTILs, CD8 + T cells and PD-L1 levels did not differ between T-stages and N-stages (data not shown), we believe that the effect of this disbalance is probably limited. In addition, tumor-intrinsic features such as tumor grade or proliferation rate might have confounded our analysis on the relation between ER expression and immune phenotype. Although we only observed minor differences in immune cell infiltration and PD-L1 expression between tumors with high grade or a high proliferation rate versus low grade or a low proliferation rate, this could have influenced our results. Our study focused on early-stage breast tumors, and therefore our conclusions cannot directly be applied to the metastatic setting. Since our series is not representative of the breast cancer population due to our enrichment of breast tumors with ER expression between 1 and 99%, it should be noted that our series is not suitable for epidemiological studies or real-world interpretation but instead our results should be considered as hypothesis-generating.

In this study, we demonstrate that early-stage breast tumors with low-positive (1–9%) and intermediate-positive (10–50%) ER expression have immunological properties with more similarities to ER-negative tumors than to ER-high tumors. Since ICB is currently only approved for TNBC, these findings highlight that the identification based on ER-negativity of breast tumors that might benefit from ICB needs revisiting. Our study encourages adequately powered subgroup analysis of patients with low-positive and intermediate ER expression in clinical trials for ICB in ER-positive breast cancer and highlights that the traditional selection based of breast cancer patients on ER expression might not be optimal for ICB treatment.

## Methods

### Study population and tissue collection

All patients presenting with primary breast cancer with ER expression on tumor cells between 1 and 50% in the Netherlands Cancer Institute between January 2011 and September 2019 were identified via the local Tumor Registry (*n* = 142). In addition, we collected a longitudinal series of tumor blocks with ER expression of 0%, 51–99% and 100%. Given that these groups are more prevalent and to roughly match the sample size of the group with ER expression between 1 and 50%, a random shorter period within the diagnosis years of 2011–2019 was used to collect the tumor blocks for the groups with ER expression of 0% (from 2016 onwards, *n* = 204), 51–99% (from 2018 onwards, *n* = 96) and 100% (Q3/Q4 2018, *n* = 89; Supplementary Fig. 1). All patients were considered to be included if they had early-stage disease, HER2-negative breast cancer and availability of tumor blocks within our institute. To ensure accurate continuous ER scoring and HER2 assessment, we only collected tumor blocks after 2011. Available archival formalin-fixed paraffin-embedded (FFPE) tumor blocks within the Netherlands Cancer Institute with known ER expression were collected in the study. When available, resection material was collected and, in case of neo-adjuvant endocrine treatment or chemotherapy, biopsies were collected. All patients with metastatic disease, or tumor blocks of local recurrences and non-invasive breast tumors were excluded. Clinical data was extracted from the local Tumor Registry from the selected patients and additional clinical data was collected directly from the patient records. Pathological characteristics, such as ER expression, PR expression, HER2 status, Ki-67 expression, and tumor grade, were obtained from the pathology reports. All histological assessments were performed in a single pathology laboratory of the Netherlands Cancer Institute in concordance with Dutch guidelines for breast cancer diagnostics^9^ including the required controls to ensure accurate receptor staining. Scoring was performed by dedicated breast cancer pathologists. The study was approved by the Institutional Review Board of the Netherlands Cancer Institute (IRBdm20-044).

### H&E and immunohistochemistry stainings

New hematoxylin and eosin (H&E) stainings were obtained from FFPE tumor blocks and tumor blocks with a tumor-cell percentage below 20% were disregarded. Immunohistochemistry of the FFPE tumor samples was performed on a BenchMark Ultra autostainer (Ventana Medical Systems). Briefly, paraffin sections were cut at 3 μm, heated at 75 °C for 28 min and deparaffinized in the instrument with EZ prep solution (Ventana Medical Systems). Heat-induced antigen retrieval was carried out using Cell Conditioning 1 (CC1, Ventana Medical Systems) for 32 min at 95 °C (CD8) or 64 min at 95 °C (PD-L1). CD8 was detected using clone C8/144B (1/200 dilution, 32 min at 37 °C, Agilent/DAKO) and PD-L1 using clone 22C3 (1/40 dilution, 1 h at room temperature, Agilent/DAKO). Bound antibody was detected using the OptiView DAB Detection Kit and slides were counterstained with Hematoxylin and Bluing Reagent (Ventana Medical Systems). Slides were scanned with a PANNORAMIC® 1000 scanner (3DHISTECH; ×40 magnification) and uploaded on SlideScore for digital assessment (www.slidescore.com). On the H&E, sTILs were assessed by an experienced pathologist (J.S.) according to established guidelines for sTIL scoring in breast cancer^59^. CD8 + T cells were scored as percentage of positive cells within the tumor-associated stromal area by the same pathologist. sTILs and CD8 scores were revised by an independent second pathologist (H.M.H.). PD-L1 expression was assessed by a dedicated breast pathologist (H.M.H.) as the combined positive score (CPS), which was defined as the number of PD-L1 positive cells (tumor cells and immune cells) divided by the total number of tumor cells multiplied by 100, as described before^60^.

### NanoString gene expression analysis

The tumor and tumor-associated stromal area was annotated on a H&E slide for subsequent RNA isolation. In case of an area of at least 8 mm^2^ and a TCP of 20%, RNA was isolated in 5-15 10 μm sections of FFPE tumor blocks (depending on area size). DNA and RNA was isolated simultaneously with the AllPrep DNA/RNA FFPE kit (Qiagen, #80234) using the QIAcube, according to the manufacturer’s instructions. The RNA concentration was measured by NanoDrop. 200 ng of RNA (or max. 12 μl in case of low concentrations) was used as input on a NanoString nCounter® platform and gene expression was assessed by the NanoString nCounter® Breast Cancer 360™ panel^61^. Kits and probes were obtained from NanoString and samples were processed by the manufacturer’s instructions. The Breast Cancer 360™ panel contains 758 genes of interest with 18 additional genes for internal reference. Only samples passing the quality control of housekeeping gene expression were included in the subsequent analysis. Genes not included in the tumor-inflammation signature (TIS) and PAM50 classification were normalized using a ratio of the expression value to the geometric mean of all housekeeping genes on the panel. Genes included in TIS and PAM50 are normalized using a ratio of the expression value to the geometric mean of the housekeeper genes used only for TIS or PAM50, respectively^62,63^. Genes not in the PAM50 signature were additionally normalized using a ratio of the housekeeper-normalized data and a panel standard run on the same cartridge or a panel standard run on the same codeset. Finally, the data was log_2_ transformed. 48 signatures capturing breast cancer biology as defined by NanoString were calculated, including TIS and molecular subtyping. Signature scores were adjusted with constants to express values in a similar range and making scores comparable across assays. The Risk of Recurrence score was log_2_ transformed to obtain values within the range of the other signatures for differential expression analysis. PAM50 subtype calling was performed as described previously^39,63^. TNBC subtypes^40^ were identified using a calculated weighted average of the luminal A and luminal B PAM50 subtype correlation and AR gene expression and the signature scores for Mammary Stemness and TIS. TNBC subtypes were called based on a set of decision rules on the aforementioned scores by NanoString.

### Gene expression analysis independent validation cohort

Gene expression data and clinical characteristics of the validation cohort were obtained directly from the primary investigators^41^. Briefly, microarray experiments (GEO accession number GSE34138) or RNA-sequencing (GEO accession number GSE192341) were performed on pre-treatment biopsies from patients with stage I-III HER2-negative breast cancer in the Netherlands Cancer Institute, treated with neo-adjuvant chemotherapy. Data from these experiments were pooled and normalized as previously described^41^. There was no overlap between samples of this independent validation cohort and the main cohort studied in this manuscript.

### Statistical analysis

Categorical variables were described as proportion of patients within each ER expression group (i.e., ER 0%, 1–9%, 10–50%, 51–99%, 100%) and differences were assessed by Fisher’s exact test. Differences between groups for continuous variables were assessed by non-parametric statistical tests: Mann–Whitney-U for differences between two groups and Kruskal–Wallis test for differences between three or more groups. Post-hoc analyses of the Kruskal–Wallis test were performed with a Dunn’s test. Differential expression analysis of signatures was performed with Qlucore Omics Explorer where the difference in group means between groups (ER 0%, 1–9%, 10–50%, 51–99%, 100%) was tested with *t*-tests for each variable. Statistical analysis was performed by SPSS statistics (IBM, version 28.0.1.0), GraphPad Prism (version 9.0.1) and Qlucore Omics Explorer (version 3.8). *P* values are unadjusted unless otherwise reported, all statistical tests were two-sided and a *p* value of <0.05 was considered statistically significant.

### Reporting summary

Further information on research design is available in the Nature Research Reporting Summary linked to this article.

## Supplementary information

Supplemental Information

Reporting Summary

Supplementary Table 1

## Data Availability

All data used for this study are included in Supplementary Table 1. Data from the validation cohort are available via GEO accession numbers GSE34138 (microarray) and GSE192341 (RNA-sequencing).
