# Supplementary material for: Immune landscape of breast tumors with low and intermediate estrogen receptor expression
Source: NPJ Breast Cancer. 2023 May 13;9:39. doi: 10.1038/s41523-023-00543-0 (PMC10182974; doi:10.1038/s41523-023-00543-0)
Supplement: Supplementary file 1 — Supplemental Information [file 41523_2023_543_MOESM1_ESM.pdf]

Supplementary figure 1

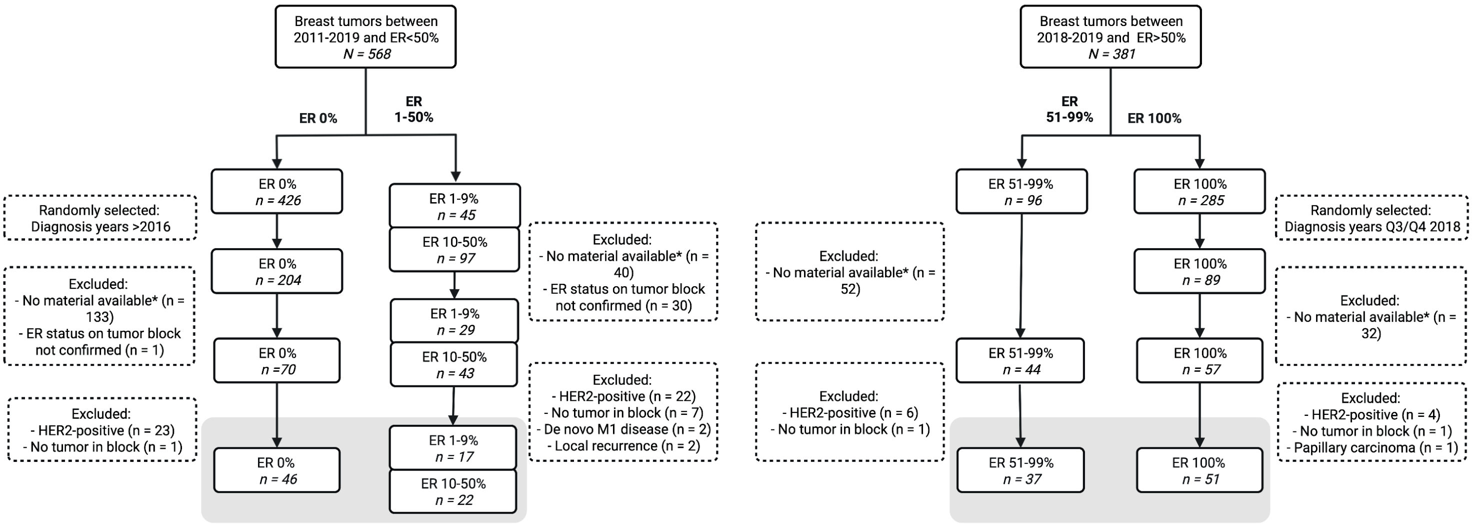

Supplementary figure 1: Flow chart of selection of tumors with different estrogen receptor (ER) expression levels. All patients diagnosed with breast tumors with ER expression between 1-50% between 2011 and 2019 were selected. For the groups with 0%, 51-99% and 100% ER expression, a random period was taken within these diagnosis years aiming for similar group sizes. Figure was created with Biorender.com. *No tumor block of untreated invasive breast tumor available within the Netherlands Cancer Institute.

Supplementary figure 2

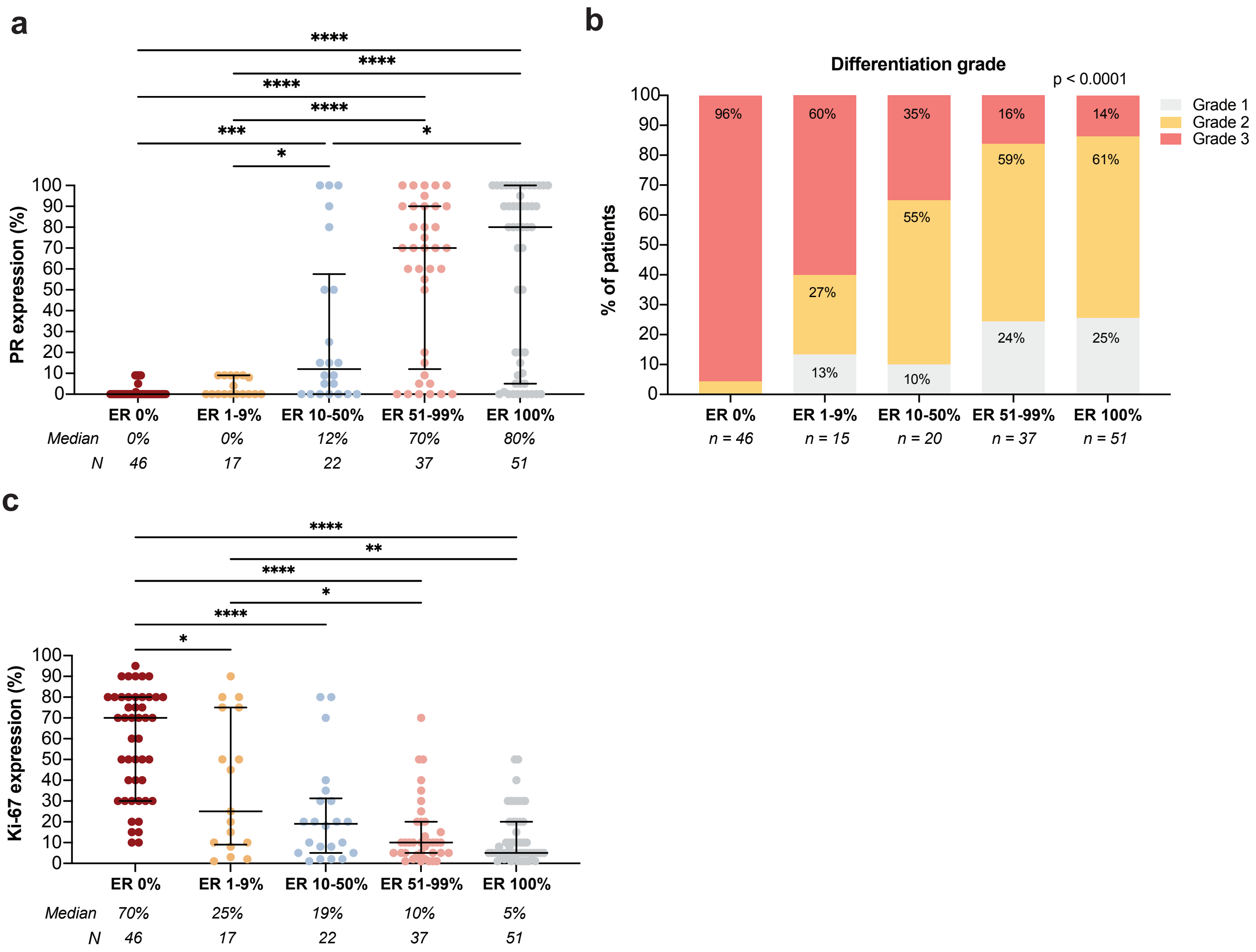

Supplementary figure 2: Intrinsic features of breast tumors with different levels of ER expression. (A) PR expression in relation to ER expression. (B) Proportion of patients per tumor grade according to Bloom-Richardson in relation. to ER expression. Numbers display percentage per group, statistics by Fisher's exact test. (C) Ki-67 expression in relation to ER expression. (A),(C) Median with interquartile range. Statistics by Kruskal-Wallis with post-hoc Dunn's test. Only statistically significant comparisons are shown. *p<0.05, **p<0.01, ***p<0.001, ****p<0.0001.

Supplementary figure 3

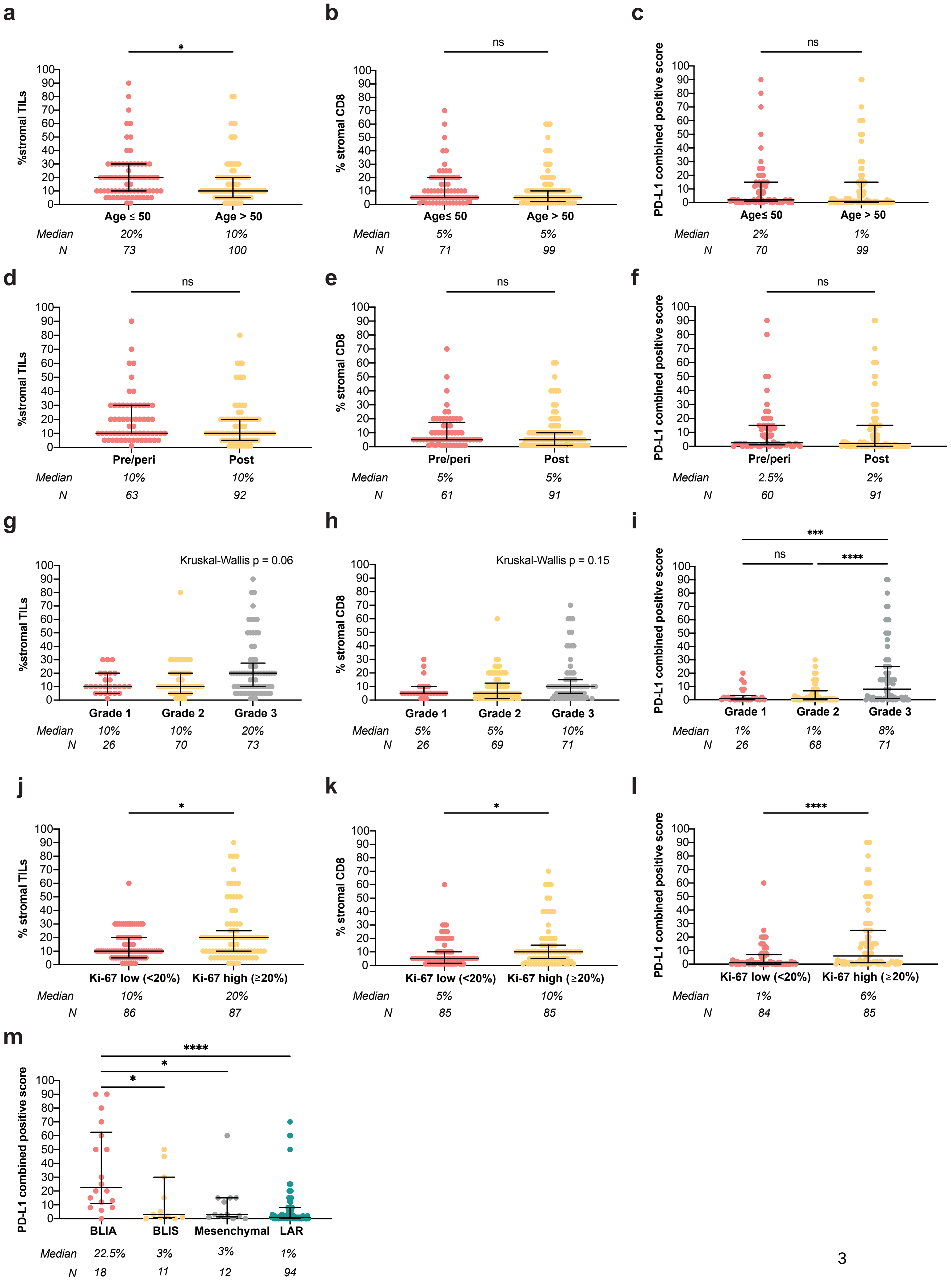

Supplementary figure 3: Immune cell composition and PD-L1 expression per age, menopausal status, tumor grade and Ki-67 expression levels. (A) Levels of stromal tumor infiltrating lymphocytes (sTILs) according to age ≤ 50 or age > 50. (B) Levels of stromal CD8+ T cells (percentage of CD8+ T cells of the stromal area) according to age ≤ 50 or age > 50. (C) PD-L1 expression, assessed as the combined positive score (CPS), according to age ≤ 50 or age > 50. (D) Levels of stromal tumor infiltrating lymphocytes (sTILs) in relation to pre- or perimenopausal status vs. postmenopausal. (E) Levels of stromal CD8+ T cells (percentage of CD8+ T cells of the stromal area) in relation to pre- or perimenopausal status vs. postmenopausal. (F) PD-L1 expression (CPS) in relation to pre- or perimenopausal status vs. postmenopausal. (G) Levels of stromal tumor infiltrating lymphocytes (sTILs) per tumor grade according to Bloom-Richardson. (H) Levels of stromal CD8+ T cells (percentage of CD8+ T cells of the stromal area) per tumor grade according to Bloom-Richardson. (I) PD-L1 expression (CPS) per tumor grade according to Bloom-Richardson. (J) Levels of sTILs in tumors with low Ki-67 expression (<20%) and high Ki-67 expression (≥ 20%). (K) Levels of stromal CD8+ T cells in tumors with low Ki-67 expression (<20%) and high Ki-67 expression (≥ 20%). (L) PD-L1 expression (CPS) in tumors with low Ki-67 expression (<20%) and high Ki-67 expression (≥ 20%). (M) PD-L1 expression (CPS) according to TNBC subtype: basal-like immune activated (BLIA), basal-like immune-suppressed (BLIS), mesenchymal or luminal androgen receptor (LAR) tumors in relation to ER expression. TNBC subtypes were assessed with the NanoString nCounter® Breast Cancer 360™ panel. For (G)-(I), (M) statistics by Kruskal-Wallis with post-hoc Dunn's test; for (A)-(F) and (J)-(L) statistics by Mann-Whitney. Only statistically significant comparisons are shown. ns (non-significant): p>0.05, *p<0.05, **p<0.01, ***p<0.001, ****p<0.0001.

Supplementary figure 4

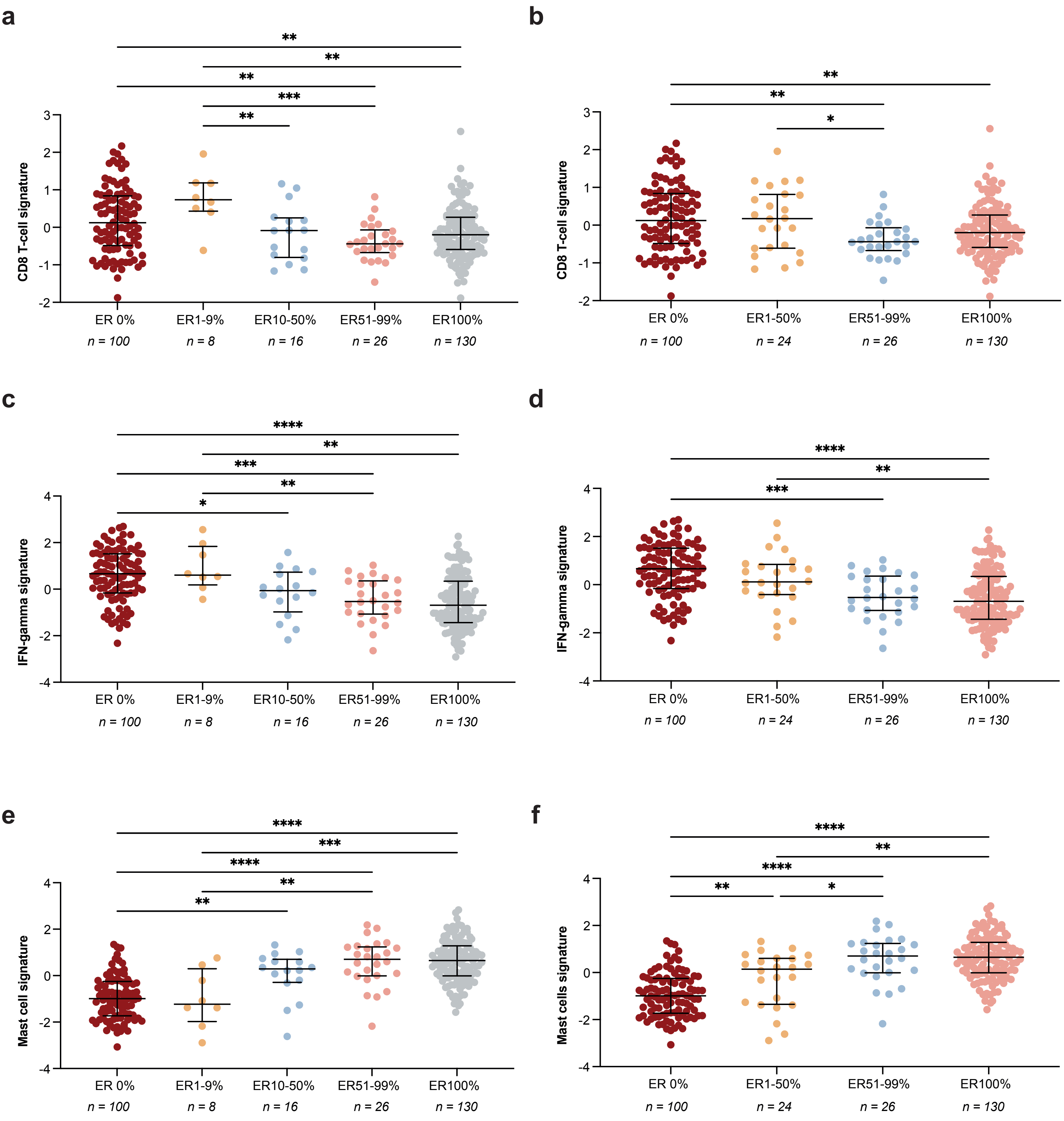

Supplementary figure 4: Expression of immune signatures in relation to estrogen receptor (ER) expression levels in an independent validation cohort. (A) CD8+ T-cell signature expression in relation to ER expression with separate groups of tumors with ER 1-9% and ER10-50%. Average expression of *CD8A, CD8B.* (B) CD8+ T-cell signature expression in relation to ER expression with a pooled group of ER1-50%. Average expression of *CD8A, CD8B.* (C) Interferon (IFN)-γ signature expression in relation to ER expression with separate groups of tumors with ER 1-9% and ER10-50%. Average expression of *CXCL9, CXCL10, STAT1*. (D) Interferon (IFN)-γ signature expression in relation to ER expression with separate groups of tumors with a pooled group of ER1-50%. Average expression of *CXCL9, CXCL10, STAT1*. (E) Mast cell signature expression in relation to ER expression with ER 1-9% and ER10-50%. Average expression of *MS4A2, CPA3, HDC, TPSAB1.* (F) Mast cell signature expression in relation to ER expression with a pooled group of ER1-50%. Average expression of *MS4A2, CPA3, HDC, TPSAB1.* (A)-(F) Median with interquartile range, statistics by Kruskal-Wallis with post-hoc Dunn's test. Only statistically significant comparisons are shown. *p<0.05, **p<0.01, ***p<0.001, ****p<0.0001.

Supplementary figure 5

a

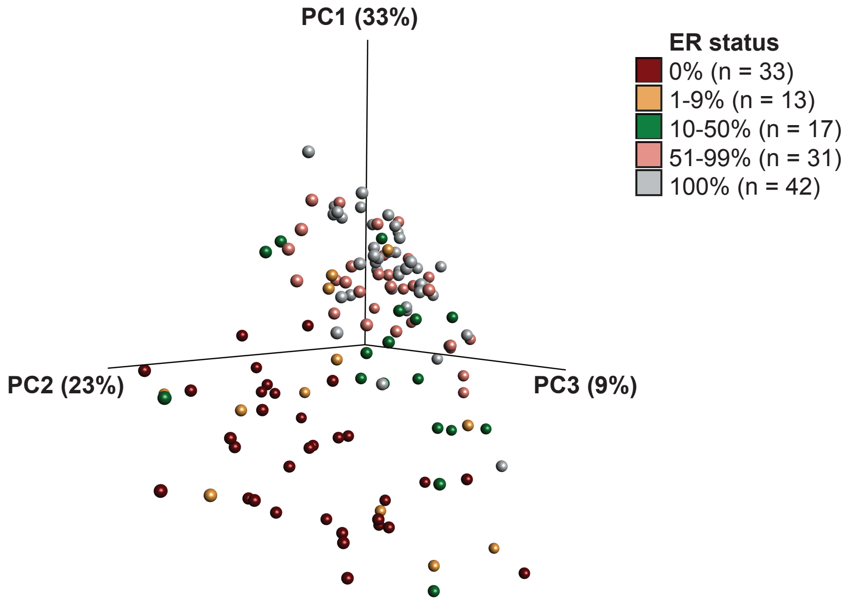

b

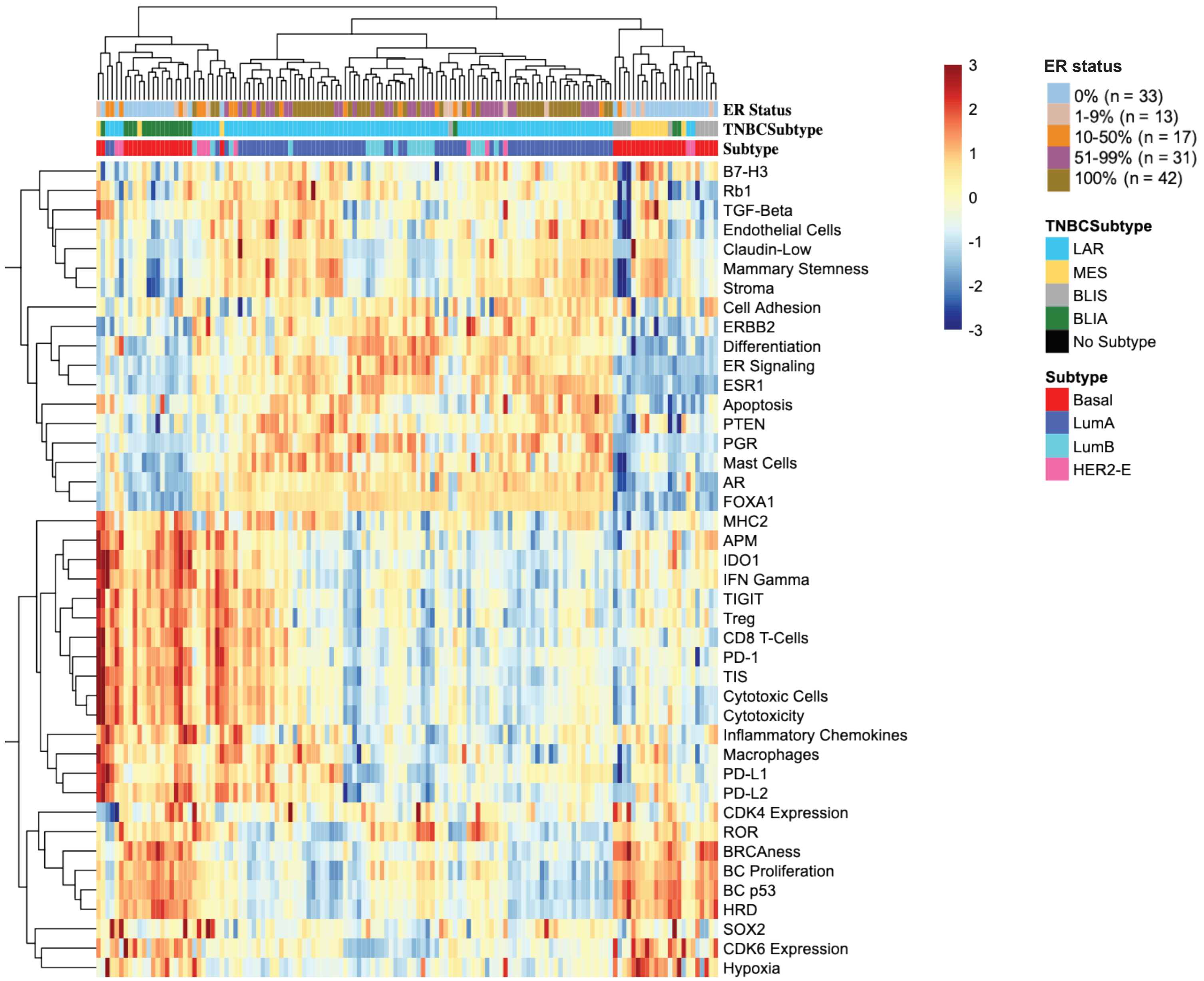

Supplementary figure 5: Unbiased analysis of NanoString Breast Cancer 360™ signatures. (A) Principal component analysis (PCA) of the NanoString nCounter® Breast Cancer 360™ panel signatures in all included tumors (n = 136). Three principal components (PC) are depicted with PC1 explaining 33% of the variance, PC2 explaining 23% of the variance and PC3 explaining 9% of the variance. Tumors are annotated by ER expression level as indicated by the legend. (B) Heatmap of normalized gene signatures across all tumors with hierarchical clustering of scaled gene signatures and samples. Samples are annotated with ER expression level, PAM50 molecular subtype and TNBC subtype.

Supplementary figure 6

a

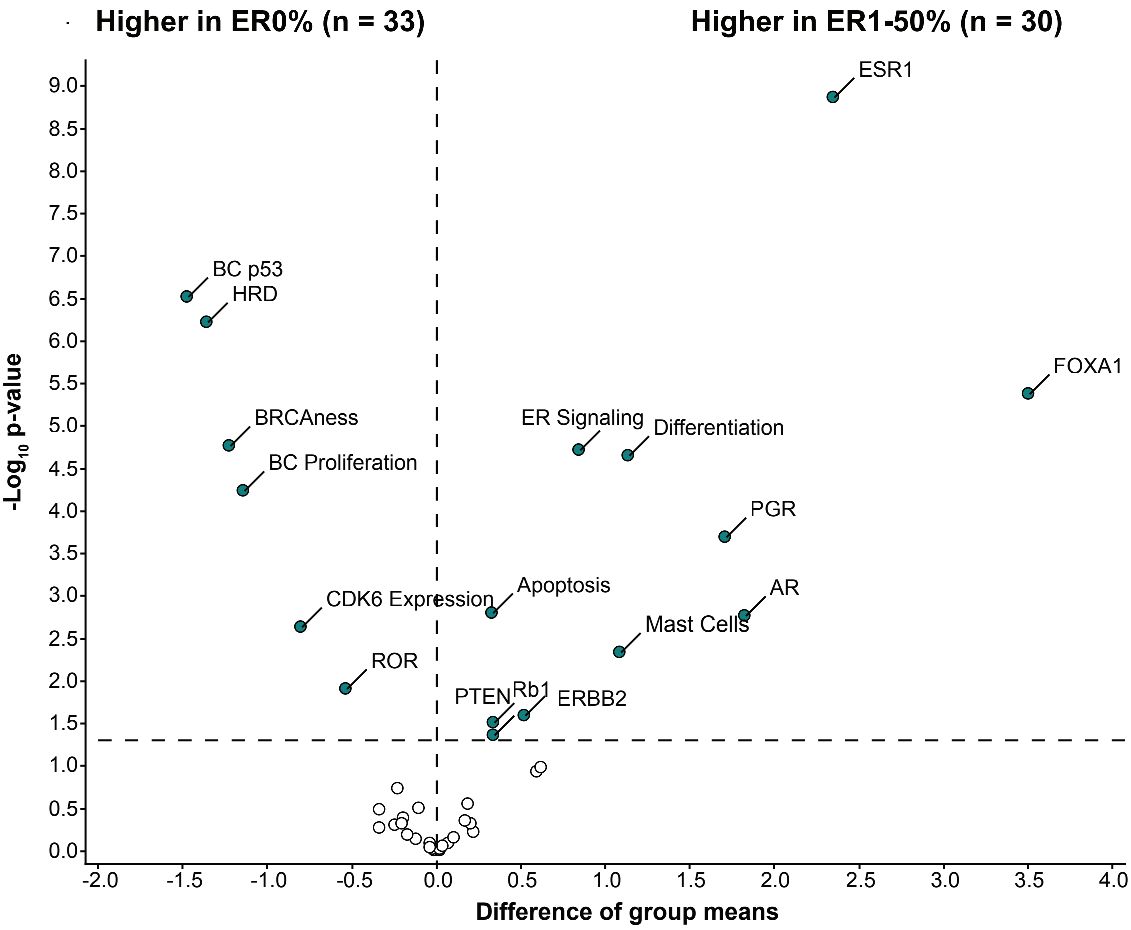

b

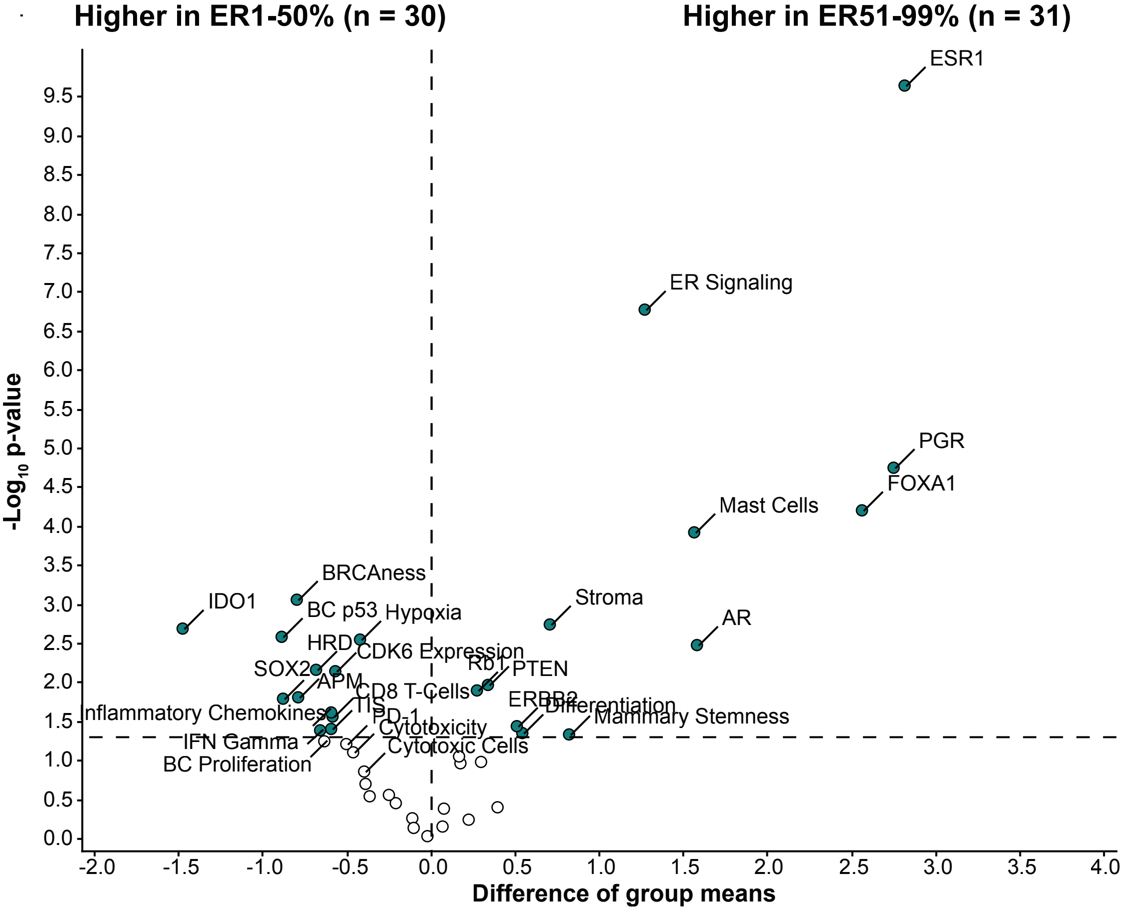

Supplementary figure 6: Differential expression of NanoString Breast Cancer 360™ signatures in pooled ER low-positive and intermediate-positive tumors. (A) Difference in gene expression of signatures between the group with 0% ER expression and the pooled group with 1-50% ER expression. (B) Difference in gene expression of signatures between the pooled group with 1-50% ER expression and 51-99% ER expression. (A)-(B) On the x-axis the difference in group means is displayed, on the y-axis the unadjusted p-value per variable by student t-tests. The vertical line indicates no change, the horizontal line indicates a p-value of 0.05.
