# Supplementary material for: Immune landscape of breast tumors with low and intermediate estrogen receptor expression
Source: NPJ Breast Cancer. 2023 May 13;9:39. doi: 10.1038/s41523-023-00543-0 (PMC10182974; doi:10.1038/s41523-023-00543-0)
Supplement: Supplementary file 2 — Reporting Summary [file 41523_2023_543_MOESM2_ESM.pdf]

nature portfolio

Corresponding author(s): Marleen Kok (m.kok@nki.nl)

Last updated by author(s): Apr 24, 2023

Reporting Summary

Nature Portfolio wishes to improve the reproducibility of the work that we publish. This form provides structure for consistency and transparency in reporting. For further information on Nature Portfolio policies, see our Editorial Policies and the Editorial Policy Checklist.

Statistics

For all statistical analyses, confirm that the following items are present in the figure legend, table legend, main text, or Methods section.

n/a	Confirmed	
☐	☒	The exact sample size (*n*) for each experimental group/condition, given as a discrete number and unit of measurement
☐	☒	A statement on whether measurements were taken from distinct samples or whether the same sample was measured repeatedly
☐	☒	The statistical test(s) used AND whether they are one- or two-sided<br>*Only common tests should be described solely by name; describe more complex techniques in the Methods section.*
☐	☒	A description of all covariates tested
☐	☒	A description of any assumptions or corrections, such as tests of normality and adjustment for multiple comparisons
☐	☒	A full description of the statistical parameters including central tendency (e.g. means) or other basic estimates (e.g. regression coefficient) AND variation (e.g. standard deviation) or associated estimates of uncertainty (e.g. confidence intervals)
☐	☒	For null hypothesis testing, the test statistic (e.g. *F*, *t*, *r*) with confidence intervals, effect sizes, degrees of freedom and *P* value noted<br>*Give P values as exact values whenever suitable.*
☒	☐	For Bayesian analysis, information on the choice of priors and Markov chain Monte Carlo settings
☒	☐	For hierarchical and complex designs, identification of the appropriate level for tests and full reporting of outcomes
☒	☐	Estimates of effect sizes (e.g. Cohen's *d*, Pearson's *r*), indicating how they were calculated

Our web collection on statistics for biologists contains articles on many of the points above.

Software and code

Policy information about availability of computer code

Data collection	Clinico-pathological data was collected from the tumor registry of the Netherlands Cancer Institute and additional data was extracted directly from the patient records. Gene expression data was obtained via the NanoString nCounter platform.
Data analysis	Gene expression was analyzed with the commercial NanoString nCounter platform.

For manuscripts utilizing custom algorithms or software that are central to the research but not yet described in published literature, software must be made available to editors and reviewers. We strongly encourage code deposition in a community repository (e.g. GitHub). See the Nature Portfolio guidelines for submitting code & software for further information.

Data

Policy information about availability of data

All manuscripts must include a data availability statement. This statement should provide the following information, where applicable:

- Accession codes, unique identifiers, or web links for publicly available datasets
- A description of any restrictions on data availability
- For clinical datasets or third party data, please ensure that the statement adheres to our policy

All data used for this study are included in Supplementary Table 1. Data from the validation cohort are available via GEO accession numbers GSE34138 (microarray) and GSE192341 (RNA-sequencing).

Human research participants

Policy information about studies involving human research participants and Sex and Gender in Research.

Reporting on sex and gender	In this study, we have included one male patient versus 172 female patients. We did not purposely selected patients based on gender but instead focused on collection material within groups of different ER expression levels.
Population characteristics	Breast tumors of a total of 173 patients with stage I-III HER2-negative breast cancer were characterized in our study. Tumor blocks were collected from biopsies before neo-adjuvant treatment or resection material in case of no treatment. As described in Table 1, age and menopausal status were equally distributed across the investigational groups with approximately 50% of patients being 50 years or younger. T stage and nodal stage were slightly imbalanced between groups and 8 patients had a germline BRCA mutation.
Recruitment	Patients were not actively recruited but retrospectively analyzed (via opt-out procedures or more recently opt-in).
Ethics oversight	The study was approved by the Institutional Review Board of the Netherlands Cancer Institute.

Note that full information on the approval of the study protocol must also be provided in the manuscript.

Field-specific reporting

Please select the one below that is the best fit for your research. If you are not sure, read the appropriate sections before making your selection.

☒ Life sciences ☐ Behavioural & social sciences ☐ Ecological, evolutionary & environmental sciences

For a reference copy of the document with all sections, see nature.com/documents/nr-reporting-summary-flat.pdf

Life sciences study design

All studies must disclose on these points even when the disclosure is negative.

Sample size	All patients were selected with ER expression between 1 and 50% and diagnosed between 2011 and 2019. A total of 39 patients had available tumor blocks in the Netherlands Cancer Institute (Supplementary Figure 1) and were used in this study. To roughly match this group size for the groups with 0%, 51-99% and 100% ER expression, we collected data from a consecutive series within a shorter frame in these diagnosis years (see Supplementary Figure 1 for details).
Data exclusions	All patients were excluded that were HER2-positive, for which ER expression could not be confirmed in the original pathology report, for which no tumor block was available for translational research or when distant metastasis were present.
Replication	This is a unique patient cohort tailored for this study and not meant to represent a real-life cohort for epidemiological studies. Our analysis can be replicated via the open access data table provided with this manuscript.
Randomization	No treatment allocations.
Blinding	Pathologists that scored sTILs, CD8+ T cells and PD-L1 expression were blinded for ER expression. No other blinding applies.

Reporting for specific materials, systems and methods

We require information from authors about some types of materials, experimental systems and methods used in many studies. Here, indicate whether each material, system or method listed is relevant to your study. If you are not sure if a list item applies to your research, read the appropriate section before selecting a response.

Materials & experimental systems

n/a	Involved in the study	
☐	☒	Antibodies
☒	☐	Eukaryotic cell lines
☒	☐	Palaeontology and archaeology
☒	☐	Animals and other organisms
☐	☒	Clinical data
☒	☐	Dual use research of concern

Methods

n/a	Involved in the study	
☒	☐	ChIP-seq
☒	☐	Flow cytometry
☒	☐	MRI-based neuroimaging

Antibodies

Antibodies used	CD8 IHC: clone C8/144B<br>PD-L1 IHC: clone 22C3
Validation	CD8 was detected using clone C8/144B (1/200 dilution, 32 minutes at 37°C, Agilent/DAKO) and PD-L1 using clone 22C3 (1/40 dilution, 1 hour at room temperature, Agilent/DAKO). All antibodies were validated for its intended use.

Clinical data

Policy information about clinical studies

All manuscripts should comply with the ICMJE guidelines for publication of clinical research and a completed CONSORT checklist must be included with all submissions.

Clinical trial registration	No clinical trial
Study protocol	No study protocol available since this is a retrospective analysis.
Data collection	Clinical data is collected in the tumor registry of the Netherlands Cancer Institute. After permission of the IRB, M.S.K., M.D. and L.V. further curated the data and enhanced the dataset with additional clinical features. Data was collected from patients diagnosed with breast cancer between 2011 and 2019 in the Netherlands Cancer Institute. Data collection and curation took place between Q2 2020 and Q3 2021.
Outcomes	Outcomes focused on immunological features of breast tumors, including sTILs, CD8+ T cells and PD-L1 expression. Additional outcomes were molecular subtype based on PAM50 and TNBC and immune-related gene expression.
